# Alarm Management in Intensive Care: Qualitative Triangulation Study

**DOI:** 10.2196/55571

**Published:** 2024-06-18

**Authors:** Lina Mosch, Meltem Sümer, Anne Rike Flint, Markus Feufel, Felix Balzer, Frauke Mörike, Akira-Sebastian Poncette

**Affiliations:** 1 Institute of Medical Informatics Charité – Universitätsmedizin Berlin, corporate member of Freie Universität Berlin and Humboldt Universität zu Berlin Berlin Germany; 2 Department of Anesthesiology and Intensive Care Medicine Charité – Universitätsmedizin Berlin, corporate member of Freie Universität Berlin and Humboldt Universität zu Berlin Berlin Germany; 3 Division of Ergonomics, Department of Psychology and Ergonomics (IPA) Technische Universität Berlin Berlin Germany; 4 Department of Rehabilitation Sciences, Research Unit Work, Inclusion and Technology Technische Universität Dortmund Dortmund Germany

**Keywords:** digital health, transdisciplinary research, technological innovation, patient-centered care, qualitative, ethnographic, ethnography, intensive care unit, ICU, intensive care, German, Germany, Europe, European, interview, interviews, alarm, alarms, intelligent, artificial intelligence, grounded theory, experience, experiences, attitude, attitudes, opinion, opinions, perception, perceptions, perspective, perspectives

## Abstract

**Background:**

The high number of unnecessary alarms in intensive care settings leads to alarm fatigue among staff and threatens patient safety. To develop and implement effective and sustainable solutions for alarm management in intensive care units (ICUs), an understanding of staff interactions with the patient monitoring system and alarm management practices is essential.

**Objective:**

This study investigated the interaction of nurses and physicians with the patient monitoring system, their perceptions of alarm management, and smart alarm management solutions.

**Methods:**

This explorative qualitative study with an ethnographic, multimethods approach was conducted in an ICU of a German university hospital. Using triangulation in data collection, 102 hours of field observations, 12 semistructured interviews with ICU staff members, and the results of a participatory task were analyzed. The data analysis followed an inductive, grounded theory approach.

**Results:**

Nurses and physicians reported interacting with the continuous vital sign monitoring system for most of their work time and tasks. There were no established standards for alarm management; instead, nurses and physicians stated that alarms were addressed through ad hoc reactions, a practice they viewed as problematic. Staff members’ perceptions of intelligent alarm management varied, but they highlighted the importance of understandable and traceable suggestions to increase trust and cognitive ease.

**Conclusions:**

Staff members’ interactions with the omnipresent patient monitoring system and its alarms are essential parts of ICU workflows and clinical decision-making. Alarm management standards and workflows have been shown to be deficient. Our observations, as well as staff feedback, suggest that changes are warranted. Solutions for alarm management should be designed and implemented with users, workflows, and real-world data at the core.

## Introduction

### Background

In intensive care units (ICUs), continuous monitoring of multiple vital signs results in a high number of alarms [1] intended to inform staff of critical patient conditions, thus ensuring patient safety [2]. However, a large proportion of alarms do not require medical intervention [3], which, along with the sheer amount of alarms, disturb patient care [4]. In addition, the desensitization of ICU staff to alarms, *“*alarm fatigue” [5-7], is a threat to patient safety as it causes slower or no reactions to alarms [8,9]. Alarm fatigue is associated with working conditions and individual staff characteristics in deteriorating alarm monitoring performance [10]. Thus, for an improved monitoring performance, an understanding of workflows and the inner setting of a unit conducting vital sign monitoring is essential.

Alarm management standards have been proven effective in reducing unnecessary alarms [11,12]. Individual alarm thresholds, tailored to a patient based on previous monitoring data, alarm logs, and the medical data stored in the patient data management system (PDMS), would further improve alarm management [13,14]. One approach to this is using artificial intelligence (AI) to integrate these data and suggest alarm thresholds for an individual patient or actions to take after an alarm [15,16]. The research project *Intelligent Alarm Optimizer for the Intensive Care Unit* (INALO) follows this approach by creating a data set with semiautomatically annotated data (ie, alarms and the reactions to it) and using machine learning to predict the probability of an alarm to be actionable or nonactionable [17].

Before introducing new procedures or technologies in a complex sociotechnical environment such as an ICU, the characteristics of this setting and the individuals working there should be assessed [18,19], especially focusing on lived everyday work practices [20]. Since many alarm reduction solution approaches known in research have failed because of being too general [5], the interaction of the ICU staff with the patient monitoring system and the practiced alarm management should be investigated.

### Objective

This qualitative study aimed:

1. to investigate the sociotechnical system ICU, with regard to the interaction of staff with the patient monitoring system and its alarms and
2. to understand the staff’s perceptions of alarm management and the potential they see in the use of AI in this context.

We aimed to address the following research questions:

1. What is the sociotechnical role of patient monitoring in the work practice of nurses and physicians of the research site?
2. How does the staff, including nurses and physicians, of an ICU of a university hospital interact with the patient monitoring system, especially their interaction with alarms?
3. What is the attitude of physicians and nurses toward intelligent alarm management systems?

## Methods

We consulted the Standards for Reporting Qualitative Research for reporting this research [21].

### Qualitative Approach and Research Paradigm

This ethnographic study follows an explorative approach with (1) observation-based data collection triangulated with (2) semistructured interviews supported by (3) a self-reported overview of activities mapped out by each participant [22-24]. The research paradigm is postpositivist. We aimed to maintain a holistic, observing perspective while acknowledging that all the collected data and the derived findings are fallible, value laden, and need to be reflected from different researchers’ perspectives [25]. The use of ethnographic methods fulfills the requirements of the postpositivist paradigm; it can create an understanding of social structures and capture the role and interaction with a technical system within those structures, revealing underlying sociotechnical relationships and patterns [26]. In order to build this comprehensive understanding, ethnographic research usually uses other methods in addition to observation [27]. Therefore, in addition to observation, semistructured interviews were conducted, and a participatory task was set for this work.

### Researcher Characteristics and Reflexivity

The interdisciplinary research team consisted of a physician with work experience in medical informatics and anesthesiology and a research focus in implementation science and patient monitoring (LM); a psychologist enrolled in the master’s program, *Human Factors*, exploring the interface between humans and technology (MS); a digital clinician scientist with work experience in medical informatics, anesthesiology, and internal medicine and a research focus on patient monitoring and alarm management (ASP); a professor of medical informatics (FB); a professor of inclusive work systems with research focus on ethnographic methods for technology design (FM); and a professor of ergonomics with a focus on human factors research in medical work environments (MF). MS was methodologically trained by FM and conducted the data collection from the etic perspective, that is, from the perspective of a person foreign to the field and the field of activity, supervised closely by FM throughout the field work. LM’s and ASP’s clinical perspectives provided a strong interdisciplinary contextualization of the data and informed the analysis. Throughout all phases of the research, FM, MS, and LM met regularly to discuss and reflect on the data collection process and preliminary findings. None of the research team members had a direct professional relationship with the research field.

### Context

The motivation for this research, along with literature evidence of alarm fatigue, was a previous study that we had conducted to identify clinical requirements of future patient monitoring. We found that staff perceived alarm management as insufficient, threatening patient safety and disturbing workflows [28].

#### Setting

The research field of this study was an ICU of a German university hospital, where up to 21 patients can be treated in nine 2-bed rooms and six 1-bed (isolation) rooms. The devices used in the patient rooms were a continuous monitoring system (Philips IntelliVue) with sensors monitoring multiple vital parameters (eg, electrocardiogram, blood pressure, temperature, oxygen saturation, intracranial pressure, and electroencephalogram); a ventilator (Dräger Evita V800); infusion pumps (Agilia connect by Fresenius Kabi); and, if needed, a temperature management system (Arctic Sun by BD) or a dialyzer (various manufacturers). A table near the patient’s bed with a computer enabled the caregivers to call up the PDMS to retrieve patient data and to document examination results. The PDMS used in this setting was COPRA 6 (COPRA System GmbH) [29], while the hospital information system used was i.s.h.med by Cerner [30]. The patient monitoring system installed on the ward included screens at the nurses’ workstations directly outside the patient rooms and in the ward pulpit, the conference room, and a medication room. These screens displayed an overview of the vital signs of patients in adjacent rooms or of all patients in the ward.

#### Intelligent Alarm Optimizer Project

This study was conducted before the implementation and testing of INALO [31]. This project aims to improve alarm management for medical personnel using AI to decrease alarm loads, therefore easing the burden of alarm fatigue. The methodological approach included integrating and annotating data from multiple sources, including the hospital-wide patient monitoring system, the PDMS, and the hospital information system.

### Access to the Field and Sampling Strategy

Access to the field was provided by the medical supervisor of this work (ASP) as well as the nursing management of the researched unit. The observations were announced to the entire ward staff by the ward manager via email, and the staff members were provided with the opportunity to address any concerns or queries they might have. A total of 12 observations were conducted by MS, each of which involved shadowing a nurse or physician for the duration of a shift (Table 1), for a total of 102 hours of observational data. The first 2 observations, shadowing both a nurse and a physician, were considered pilot observations. Their purpose was to become familiar with the environment of an ICU, test the methodology of observation and interviewing, and adapt the interview questions. The data from pilot observations were not used for later analysis.

**Table 1:** Number of observations divided by shift and profession and level of expertise, respectively^a^.

Profession and level of experience	Early shift	Late shift	Night shift
Nurse (1.5-30 years of work experience in the ICU^b^)	3	2	1
Resident physician (1-2 years of work experience in the ICU)	2	1	—^c^
Senior physician (7 years of work experience in the ICU)	1	—	—

^a^One participant was shadowed twice.

^b^ICU: intensive care unit.

^c^Not applicable.

The 5 nurses shadowed were suggested by the ward manager and scheduled with MS for different observation days after they had given written consent to participate in the study. Physicians were contacted by MS partly with the help of the medical supervisor or directly in the course of a field observation. In total, 4 physicians agreed to participate in the study.

The aim was to shadow a sample of staff members representative of the whole team, thus covering the greatest possible variety of perspectives in the research. Nurses and physicians of different ages and experience levels in an ICU were shadowed in all possible work shifts (early, late, and night shifts). The number of accompanying nurses and physicians per shift is listed in Table 1, where the experience level of working on an ICU is shown as the duration of work in intensive care.

### Ethical Considerations

The study was approved by the ethics committee of the Charité Universitätsmedizin Berlin (EA4/218/20). Field notes and transcripts were anonymized or, if complete anonymization was not possible, pseudonymized. The data are stored on an internal institutional server, encrypted so that only the research team had access to it. Audio recordings were deleted after transcription. All staff members shadowed and interviewed for this study were informed about the research project and its aims and gave their written consent to participate. There was no compensation given to the staff members who were shadowed or interviewed.

### Data Collection Methods and Instruments

#### Field Observation

The main data collection method in this study was field research in the form observation. In ethnographic research, the observation part varies by the degree of participation of the researcher in the field [32]. The setting of the field observations and the nonmedical background of the researcher suggest a nonparticipatory observer role (shadowing), in which events are observed in the background without involvement in any work processes. This methodology has been previously used in clinical settings [33-36]. Although the researcher observed the field from the background most of the time, she was also involved in conversations during the shifts by the shadowees. This gave her the opportunity to ask clarifying questions, which was beneficial in gaining insights. The observations were performed from April to October 2021. Breaks of up to 3 months were taken for interim analysis of the data. This allowed for the readjustment of focus for the remaining observations and the evaluation of inductive thematic saturation [37], ultimately leading to the determination of the number of field observations as 7.

#### Semistructured Interviews

The interviews serve to explore the nonobservable topics relevant to the research question. They add to the observation method in the sense of methodological triangulation [22]. In particular, reactions to the planned AI-based INALO system and specific inquiries about the handling of the monitoring and its alarms were assessed. By not including the explicit term *AI* in the interview guide, the goal was to avoid potential biases or preconceived notions associated with the term, allowing for a more neutral and open interview discussion. *AI* was considered a polarized term, evoking personal opinions, as people may have subjective and varied views on the topic [38-40]. Consequently, the interviewees were provided with an explanation of the functionality of the INALO system, leaving out the specification *AI* (refer to question 5 in the interview guide in Textbox 1). The interview guide was developed through discussions of the research team and informed through the first 2 pilot observations

**Interview guide.**

**Questions**

- Are there certain situations in which the sounding of an alarm is particularly inconvenient for you? Which ones?
- What role does managing alarms and especially their thresholds currently play during your work?
- How do you know it is time to adjust the alarm thresholds of certain parameters?
- When do you take the time to adjust an alarm threshold?
- Imagine a system, in addition to patient monitoring, that controls the alarm thresholds for oxygen saturation, arterial blood pressure and heart rate. If it registers that the current thresholds are no longer suitable for the patient in question, it suggests suitable alarm thresholds. What do you think about such a system?

In total, 7 interviews were performed individually with the 7 shadowed staff members (Table 1) after the end of a work shift in an open space to minimize outside distractions. The mean length of the interviews was 37:57 (range 56:22-15:05) minutes. We considered data saturation [37] including notes from field observations and interview transcripts. It was achieved after 6 field observations and, correspondingly, 6 interviews, which included early, late, and night shifts as well as nurses, resident, and senior physicians.

#### Participatory Task

In addition to the observation and the interview, following the triangulation strategy [41], a participatory task was conducted with the study participants. This helped gain insight into the representation of patient monitoring in the daily work routine and workflows of ICU staff members.

In a classic note-and-pen task, participants were asked to draw a pie chart of all the thoughts and tasks they think about during a work shift, with each piece of pie representing a thought, a task, or a task area. The size of the pie pieces was intended to show how the respondent’s cognitive resources were divided during a shift. The participants were requested to prioritize the tasks that they had visualized based on their subjective perception. This was done to compare the estimated time allocated to each task in their workday with its perceived importance. The goal was note only to obtain indirect evidence on the importance of monitoring and alarms but also to understand the work routine in general from the respondent’s perspective.

### Data Collection Instruments and Technologies and Data Processing

Handwritten field notes were prepared following the methods of Emerson et al [42], and the date, location, and content of the observation were taken and promptly consolidated in MAXQDA (VERBI GmbH) [43] after each observation session. Interviews were recorded and transcribed according to the minimal transcript based on GAT 2 [44] and consolidated in MAXQDA. The participatory task was recorded in order to be able to use follow-up questions about the processing of the task as well as incidental comments for the later analysis.

### Data Analysis

For inductive analysis, a grounded theory approach was followed [45]. The consolidated field notes and transcribed interview data from 6 observations were analyzed in MAXQDA 2022 and coded line by line in a first step, deriving thematic codes from the data while trying to neglect any relationship of the codes to the overarching research questions. In a second step, memoing was started to extract meaning from the data and to create and map an initial overview of themes [46]. This overview represented a preliminary code system after 6 observations. After all the gathered data were collected, coded, and tagged with open memos, the result consisted of 1336 coded data sections assigned to 47 parent codes and 70 thematically subordinate codes and tagged with 207 memos. Memos (ie, field notes with headlines) were organized thematically and considered in the context of their associated data sections (eg, interactions with the monitoring systems and alarm management). To ensure this thematic clustering according to the memo headline was rightful, we went back into the raw data (field notes) to evaluate whether the groups of memos with a similar headline could indeed be grouped together based on their raw data points. This helped to reevaluate and refine the derived themes from the memos (eg, integrating memos for more specific details and adding key points from field notes to memos from interviews and vice versa). When analyzing the data collected through the participatory task, the recorded topics were written down, divided according to occupational group (nurses and physicians), and their occurrence was counted. As a result, an overview of superficial and seemingly less relevant task areas was created. The resulting themes from memos, codes, and topics from the participatory task were summarized and put in writing.

### Techniques to Enhance Trustworthiness

Regular research meetings took place, where LM and MS discussed the findings and reflected on them. The code system (Multimedia Appendix 1) and memos were checked by both researchers under the supervision of FM. The combination of a psychologically trained Human Factors graduate student (MS) together with an expert in ethnographic research for work systems design (FM) and a physician with professional experience in an ICU and expertise in qualitative methods and implementation science (LM) was chosen to achieve the best balance of perspectives and topic prioritization. Interdisciplinary approaches are important to leverage the potential of research on the intersection of human-computer interaction, information systems, and health [47]. In addition, the multimethod study design with triangulation of complex data allowed for an increased credibility and trustworthiness of the results.

## Results

### Overview

The following 3 topics were identified from the data, following the research questions:

- the perception of the role of monitoring in the ICU
- the management and communication of vital sign limits (*dealing with alarms*)
- wishes and concerns regarding the intelligent alarm management system (eg, INALO)

An overview of nurses’ and physicians’ perceptions can be found in Table 2.

**Table 2:** Perceptions of monitoring and alarm management divided by professions.

Categories	Nurses	Physicians
The role of monitoring in the intensive care unit	Use monitors at the bedside Direct monitoring of vital signs at the screens	Use monitors in the pulpit Use nurses as monitoring filter
Dealing with alarms	Subconscious adaptation to alarm patterns First reaction to alarms Implement alarm thresholds	Subconscious adaptation to alarm patterns Set and adjust alarm thresholds
Intelligent alarm management	Concerned about the system’s functionality Highlighted the importance of understanding the system’s operational principles Concerned about excessive confidence	Perceived as positive for lowering alarm burden Perceived as valuable for new patients in the ward or upon returning from leave

### The Perception of the Role of Monitoring in the ICU

Continuous monitoring of vital signs is an essential component of intensive care management of critically ill patients and a ubiquitous part of the ICU under study. According to a senior physician, even a short period without monitoring would be “grossly negligent.”

The nursing staff primarily used the monitors located directly at the patient’s bedside (ie, *direct* monitoring), as well as the overview screens at their workstations outside the patient rooms. During lengthy nursing activities in a room, it was observed that nurses used the monitoring system’s function to display other patients’ vital sign data on the screen of the bed where they were busy. Due to the larger number of patients under their care, physicians relied on the feedback from the nursing staff in the event of a critical situation (nurses as *monitoring filter*). They also paid closer attention to alarms on the overview monitors at the pulpit, and it was important that alarm thresholds were well set so that potentially life-threatening situations did not go unnoticed for long:

Especially when patients deteriorate, you notice that very often because the alarms are triggered all the time...These alarms...also signal something to us[...], without us seeing the patient directly[...], and if [the thresholds] were always set lower and it simply no longer alerts me, I’d only notice it when it’s really bad or too bad or the catastrophe is there, and I wouldn’t want that.

Interview, physician

When making therapy decisions, physicians considered the target values for specific parameters that had to be adhered to, based on therapy plans. Continuous monitoring of vital signs was crucial for physicians in ensuring that these target values are met.

Although monitoring was used in various areas of activity, it was not considered a central activity in and of itself. In the participatory task, monitoring was not mentioned by anyone. Alarms occurred in 2 of the 5 participatory tasks completed. However, when asked to indicate the role of monitoring in all the tasks written down, interviewees assigned monitoring to most of them (Multimedia Appendix 2).

### Dealing With Alarms

The predominant observation regarding the handling of alarms was that they were directly paused or completely ignored, unless it was a red alarm. Often, there was no further reaction or intervention following an alarm.

However, as described in the previous section on monitoring, even an alarm that did not require intervention could provide helpful information about the progress of an individual patient’s condition. Both physicians and nurses showed a reliance on the alarms’ feedback to be passively informed of the change in their patients’ condition (subconscious adaptation to alarm patterns). In contrast, the alarms interfered with their work:

[...]as you may have noticed, and if this alarm comes up all the time...I actually look at it [the overview monitor] a lot. That disturbs me in my regular workflow.

Interview, physician

The alarm thresholds of vital parameters were set and adjusted either based on a patient’s admission to the ward or return from the operating room or based on evaluations of the thresholds during the course of therapy. In the case of newly admitted patients, the threshold values were determined directly on admission, with the parameters being set according to standard values or having to be determined individually depending on the clinical picture. Nurses, in consultation with physicians, adjusted alarm thresholds according to the patients’ condition to avoid unnecessary alarms. The decision to adjust thresholds varied based on the nurse’s experience and familiarity with the patient. However, these informal procedures sometimes led to inappropriate thresholds on the next shift, as they were not always updated in the COPRA PDMS [29]:

...I come to the ward, and I don’t know the patient. COPRA is binding, what is documented there should be correct and implemented at the bedside. If you’re lucky, it says everything about which blood pressure values are desired...You have to ask about all that [if it is not entered correctly] and that’s sometimes a bit annoying, until you find out which doctor is responsible for your room, where he is and what his telephone number is. It doesn’t have to be like that.

Interview, nurse

Ideally, the determined threshold values were reevaluated daily in the morning shift. Often this was not the case due to a lack of time. Rather, this reevaluation only happened, if a new test result or diagnosis was found or in the case of disturbing, conspicuous, and unnecessary alarms. The chain of communication regarding the change of alarm thresholds from their determination to their implementation in monitoring ran hierarchically downwards, from senior physicians to assistant physicians to nursing.

If the alarm of a certain parameter would continue despite intervening measures or if no measures had been necessary at all, the alarm thresholds would be adjusted. Physicians and nurses frequently sought each other out for direct and immediate instructions. The official way of issuing instructions was via the COPRA PDMS, but according to staff, this was often too time-consuming, with delays for actual implementation:

And this adjustment [of the thresholds]...doesn’t take place so much on this piece of paper, but rather on the patient himself, that I say, ok, we have to change the threshold here[...], then you discuss it next to the device itself with the nursing staff.

Interview, physician

### Staff Perceptions of Intelligent Alarm Management

There were mixed reactions regarding an automated system to improve alarm management on the part of the nursing staff interviewed. Some expressed reservations, while others were open to the system described but said it would have to work well. There were concerns about the functionality of an intelligent system. The importance of understanding how such a system operates was highlighted. Staff members argued that currently a lot of responsibility was being handed over to technology anyway and that it would be more appropriate to be cautious about overconfidence:

I probably rely more on my own experience and won’t put it in the hands of a machine that I don’t know how it’s programmed, what kind of things it reacts to, or what it takes as a basis for the recommendations. That would make a lot of sense for young colleagues who don’t have a lot of experience yet, but you end up relying more on your own experience.

Interview, nurse

The patients, the clinical pictures, and that’s all so individual and different that I can’t imagine that a machine can [suggest thresholds].

Interview, nurse

Second, the statement was made that with clinical experience, one would notice when a threshold should be changed, and therefore, no system would be needed to do so:

I know already by my experience that I must intervene starting from certain values...you observe the patient, you observe the monitor and you already see...that you have to do something.

Interview, nurse

Thus, staff members saw the added value of an intelligent alarm system for less experienced colleagues and for leasing nurses, as the former would not yet have developed a sense of threshold assessment and the latter would not be familiar with patients. In addition, staff members saw a benefit in the use of the system to ensure regular evaluation and adjustment of alarm thresholds, preventing them from being forgotten.

Physicians reported that any solutions leading to a reduction in alarms would be welcome due to frequent alarms disturbing their concentration during work, especially in the ward pulpit:

[...]If it leads to the fact that the alarms are optimized, then I think it is already a relief, because I get fewer false alarms and thus, I can do the rest of the work more concentrated. So, that would already be an advantage. Certainly, it doesn’t take away my cognitive work whether these alarms are, so to speak, suitable or not, I have to do that, but that’s also my job.

Interview, physician

Finally, the advantage of such a system was observed by physicians for patients who were new to the ward and whose condition they did not yet know. The physicians said that it was also helpful to receive threshold suggestions when returning to the ward after a leave of absence, as they also needed to reacquaint themselves with patients.

## Discussion

### Principal Findings

Patient monitoring is an integral part of the work routine in the ICU. However, standards for working with the system were not implemented in clinical routine in the studied ICU. Alarm management is one of the core interactions with the monitoring system. Most alarms were confirmed without a reaction or intervention. The setting and adjusting of alarm thresholds were performed upon the arrival of a patient in the ICU or over the course of treatment by (senior) physicians and implemented by nursing staff. Perceptions of an intelligent system to suggest alarm thresholds varied: physicians saw potential advantages in a relief of the flood of (nonactionable) alarms by individualized alarm thresholds. In contrast, both nurses and physicians were skeptical about the capability of an automated system to perform the complex task of interpreting alarms and suggesting thresholds, encompassing the integration of different data and information. The importance of knowing how such a system worked internally and how it took decisions was highlighted.

### Guidelines for Patient Monitoring and Alarm Management in the ICU

This work highlights the essential role of vital sign monitoring in the daily routines of all health care professionals in the ICU. Yet, to the authors’ knowledge, interprofessional, systematic standard, and evidence-based guidelines for patient monitoring in clinical practice remain limited. Alarm management, a fundamental aspect of interacting with monitoring systems, warrants special attention, since alarm overload in ICUs hampers adequate response by personnel [48]. The American Association of Critical Care Nurses published recommendations for clinical alarm management, focusing on personalization of alarms and interprofessional alarm management strategies and highlighting the potential of smart alarms [49,50]. Our research supports the need for a wider clinical implementation of such recommendations, as we saw that a majority of alarms are disregarded or confirmed without any medical response or intervention [51,52].

### Take Aways for Future Alarm Management

It is crucial to understand user perspectives when developing intelligent (AI-based) systems for alarm management in intensive care medicine [49]. The following aspects should be considered.

- Skepticism exists among staff members regarding the ability of an intelligent system to integrate diverse data formats and information to effectively interpret alarms and propose suitable thresholds. It might stem from a lack of knowledge and understanding of the internal workflows and decision-making processes of such systems. The potential benefits of integrating intelligent systems to automatically suggest personalized alarm thresholds and alleviate alarm fatigue [16,53] were acknowledged by physicians in our study.
- Standardized workflows for alarm management were not existent, and alarm thresholds and their adaptation were communicated irregularly and in a variety of ways. Well-defined and clearly communicated standard operating procedures (SOPs) for alarm management could address some of the challenges faced by health care professionals in the ICU [54,55]. Intelligent alarm management solutions should be implemented in clinical environment with well-established alarm management SOPs.
- Alarm management was performed mainly after new test results for the patients were received, a diagnosis was confirmed, or when the alarms were frequent and obviously unnecessary. This indicates that the data used for future alarm management systems based on AI need to mirror highly individual and complex medical conditions. Patients and clinical routines can differ even for various wards in the same clinic.

Extrapolating from these findings, we advise the following for ICU AI projects.

- For a truly effective implementation of AI systems, the ICU staff must be integrated in the design and implementation process, as well as possess adequate AI literacy. Encouraging and providing training to understand and use AI can empower ICU staff to embrace AI technologies confidently.
- As we need standards for alarm management workflows today, standardization is all the more essential in the context of integrating intelligent alarm management (AI-based) technologies. Significant workflow changes could be evoked and need to be considered by ICU leaders and implementation managers.

### Limitations

In this study, the research focus lay on a single setting (ICU), which was suggested as a methodological approach by Wilken et al [5], allowing for an in-depth analysis of the contributing factors to an ICU’s alarm management and dealing with the patient monitoring system. In addition to the stated benefits of this approach by the authors, it comes with limitations. On the one hand, the focus on a single ICU’s cultural peculiarities restricts the transferability of the derived recommendations to other settings. The results and methods may serve, however, as inspiration and study protocol for evaluating an ICU’s alarm management. By contrast, following a qualitative research approach, the number of shadowees and the number of shadowing days had to fit in a reasonable time schedule for the research conduction. The unit manager’s suggestion of shadowees may have introduced a selection bias, despite being informed about the goal of diverse perspective shadowing. The results presented here are therefore only an excerpt of the reality in the ICU, which must be taken into account when interpreting them.

### Conclusions

Our study highlights that interactions with the patient monitoring system and its alarms are a core part of tasks and workflows in the ICU. Alarm management tasks are performed based on ad hoc responses to clinical events; responsibilities are not well defined, and there is no standardized workflow or an SOP. Staff members were not satisfied with the current alarm management, which emphasizes a need for standard and clinician-centered guidelines in this field. Establishing SOPs for configuring and responding to alarms and considering local patient and workflow characteristics can streamline tasks and enhance the overall efficiency of care delivery. Systems that enable an intelligent alarm management to reduce alarm fatigue among staff members should be designed to make understandable and traceable suggestions, while health care professionals should be empowered to use them meaningfully through digital health literacy. By establishing these standards and thoughtfully incorporating AI into clinical workflows, health care institutions could enhance patient safety and relieve staff and patients from alarm-induced stress. To explore this effect on outcomes, more research in this field is needed.

## Appendix

Multimedia Appendix 1 Codes and subcodes derived during data analysis from field notes, interview documentation, and documentation of the participatory task.

Multimedia Appendix 2 Indicated activities of a physician and their perceived proportion of the workday adapted from the results of the participatory task performed by a physician shadowee. The tasks with monitoring influence are indicated with the monitor icon.

## Abbreviations

AI: artificial intelligence
ICU: intensive care unit
INALO: Intelligent Alarm Optimizer for the Intensive Care Unit
PDMS: patient data management system
SOP: standard operating procedure

## Data Availability

The data sets generated and analyzed during this study are not publicly available because of data privacy; however, they are available from the corresponding author (LM) upon reasonable request.
