# Supplementary material for: Alarm Management in Intensive Care: Qualitative Triangulation Study
Source: JMIR Hum Factors. 2024 Jun 18;11:e55571. doi: 10.2196/55571 (PMC11220431; doi:10.2196/55571)
Supplement: Multimedia Appendix 1 [file humanfactors_v11i1e55571_app1.docx]

**Codesystem**

| Codes and Subcodes | Number of coded segments |
| --- | --- |
| 1 Discordant knowledge | 1 |
| 2 Dealing with patients | 1 |
| 3 Workflow of nursing care | 7 |
| 4 Establishment of novel technologies and knowledge translation | 5 |
| 5 Contrast ICU and general ward | 3 |
| 6 Hospital organization | 3 |
| 7 Lifelong learning | 1 |
| 8 Disease patterns | 6 |
| 9 Hierarchy | 2 |
| 10 Reflection | 4 |
| 11 Setting | 1 |
| 11.1 Miscellaneous on the ward | 2 |
| 11.2 Shift differences | 9 |
| 11.3 Ergonomics | 10 |
| 11.4 Architecture of the ward | 2 |
| 11.5 Structure of rooms | 6 |
| 11.6 Noise background | 15 |
| 11.7 Atmosphere | 6 |
| 12 Experience | 6 |
| 13 Manual things | 8 |
| 14 AI system | 0 |
| 14.1 Cognitive performance despite AI | 1 |
| 14.2 Interaction with AI | 4 |
| 14.3 Understanding AI | 2 |
| 14.4 Understanding functionality of the system | 5 |
| 14.5 AI system - trust | 8 |
| 14.6 Imagination of an AI system | 6 |
| 14.7 Opinions on AI system | 6 |
| 14.8 Needs regarding functionality | 15 |
| 15 Communication | 0 |
| 15.2 Dealing with each other | 6 |
| 15.3 Communication chains | 8 |
| 15.4 Communication with patients | 3 |
| 15.5 Communication physicians – nurses | 60 |
| 15.6 Inefficient communication | 1 |
| 15.7 Communication cross-clinic / cross-ward | 17 |
| 15.8 Communication among physicians | 12 |
| 15.9 Behandlunsgplanung | 1 |
| 16 Workflow | 29 |
| 16.1 Telephones | 12 |
| 16.2 Morning conference | 5 |
| 16.3 Patient examination and treatment | 6 |
| 17 Differences physicians / nurses | 8 |
| 18 Characteristics of work setting | 2 |
| 18.1 Ward culture | 1 |
| 19 Ward organization | 8 |
| 20 Dealing with uncertainty | 1 |
| 21 Education | 24 |
| 22 Worklfow physicians | 15 |
| 22.1 Role senior physician | 2 |
| 22.2 Workflow senior physician | 29 |
| 23 Ward rounds | 25 |
| 23.1 Monitoring in ward rounds | 18 |
| 23.2 Nurses in ward rounds | 8 |
| 23.3 Topics in ward rounds | 3 |
| 23.4 Technology in ward rounds | 4 |
| 24 Responsibility | 16 |
| 25 Interventions | 27 |
| 26 Usability Issues | 20 |
| 27 Social roles | 16 |
| 28 Events | 9 |
| 29 Role of technology | 14 |
| 30 Individual differences in dealing with tasks | 1 |
| 31 Distraction | 3 |
| 31.1 Distraction because of alarms | 2 |
| 32 Trust in Technology | 36 |
| 33 What is said vs. what is meant | 1 |
| 34 Monitoring of patients | 46 |
| 34.1 Preparing patients for operations | 2 |
| 34.2 Parameter | 9 |
| 34.3 Specific requirements on the ward | 2 |
| 34.4 Role of monitoring | 11 |
| 34.5 Mobile monitoring | 1 |
| 34.6 Meaning of thresholds | 7 |
| 34.7 Handling of monitoring | 34 |
| 34.8 Cross-room monitoring | 22 |
| 34.9 Wanting to see patients with your own eyes | 15 |
| 34.10 Monitor is disturing | 7 |
| 35 Break | 14 |
| 36 Colleagues help each other | 16 |
| 37 Digitalization | 12 |
| 38 Documentation | 42 |
| 38.1 COPRA | 15 |
| 39 Task organization | 33 |
| 40 Alarms | 46 |
| 40.1 Alarm effects | 1 |
| 40.2 Assessment of alarms | 2 |
| 40.3 Alarms that disappear by themselves | 2 |
| 40.4 Cause of alarm | 6 |
| 40.5 False/non-actionable alarm | 7 |
| 40.6 Threshold communication | 29 |
| 40.7 Threshold management | 27 |
| 40.7.1 Decision pathways | 7 |
| 40.8 Alarm on the screen | 6 |
| 40.9 Adapt thresholds | 43 |
| 40.10 Alarm management | 33 |
| 40.11 unsuitable situation for alarm | 26 |
| 40.12 Beeping | 9 |
| 40.13 Reactions to alarms | 53 |
| 40.14 Tolerating alarms | 8 |
| 40.15 Cause of alarm avoidable | 5 |
| 41 Technology | 7 |
| 41.1 Connection to the real world | 1 |
| 41.2 Wishes | 7 |
| 41.3 Technology-positive | 4 |
| 41.4 Technology-skepticism | 3 |
