# Supplementary figures and images for: Alarm Management in Intensive Care: Qualitative Triangulation Study
Source: JMIR Hum Factors. 2024 Jun 18;11:e55571. doi: 10.2196/55571 (PMC11220431; doi:10.2196/55571)

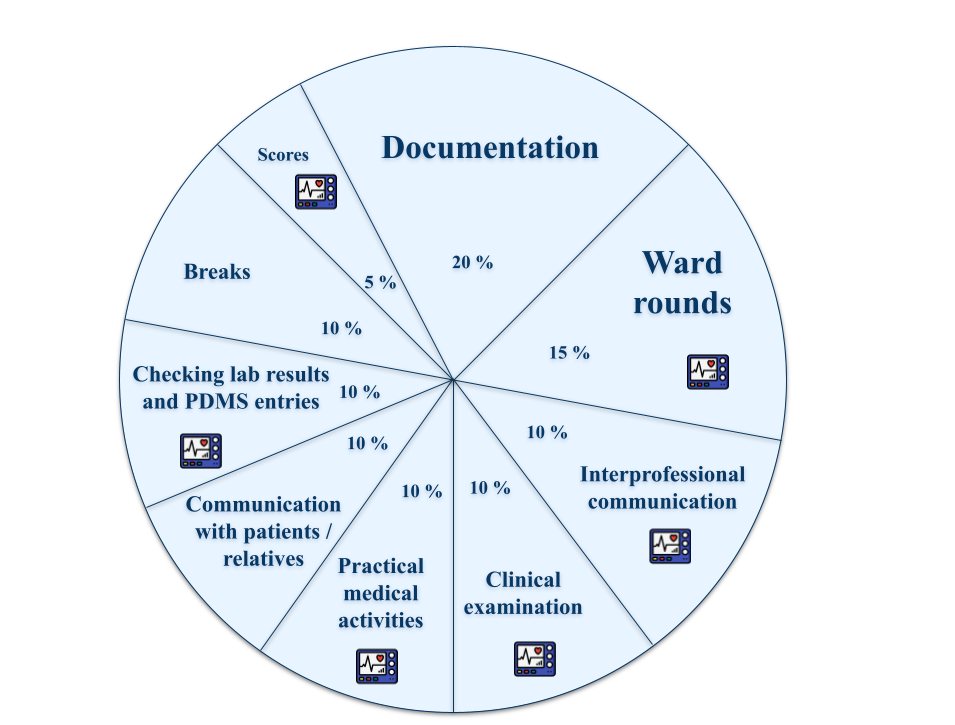

Supplement: Multimedia Appendix 2 [file humanfactors_v11i1e55571_app2.png]
